# Integrated Drug Mining Reveals Actionable Strategies Inhibiting Plexiform Neurofibromas

**DOI:** 10.3390/brainsci12060720

**Published:** 2022-05-31

**Authors:** Rebecca M. Brown, Sameer Farouk Sait, Griffin Dunn, Alanna Sullivan, Benjamin Bruckert, Daochun Sun

**Affiliations:** 1 Medicine, Hematology and Medical Oncology, Neurosurgery, The Mount Sinai Hospital, New York, NY 10029, USA; rebecca.brown@mssm.edu; 2 Department of Pediatrics, Memorial Sloan Kettering Cancer Center, New York, NY 10065, USA; faroukss@mskcc.org; 3 Department of Cell Biology, Neurobiology and Anatomy, The Medical College of Wisconsin, Milwaukee, WI 53226, USA; gdunn@mcw.edu (G.D.); arsullivan@mcw.edu (A.S.); bbruckert@mcw.edu (B.B.); 4 Department of Pediatrics, The Medical College of Wisconsin, Milwaukee, WI 53226, USA; 5 Cancer Center, The Medical College of Wisconsin, Milwaukee, WI 53226, USA

**Keywords:** plexiform neurofibromas, neurofibromatosis type 1, drug screening, gene network

## Abstract

Neurofibromatosis Type 1 (NF1) is one of the most common genetic tumor predisposition syndromes, affecting up to 1 in 2500 individuals. Up to half of patients with NF1 develop benign nerve sheath tumors called plexiform neurofibromas (PNs), characterized by biallelic NF1 loss. PNs can grow to immense sizes, cause extensive morbidity, and harbor a 15% lifetime risk of malignant transformation. Increasingly, molecular sequencing and drug screening data from various preclinical murine and human PN cell lines, murine models, and human PN tissues are available to help identify salient treatments for PNs. Despite this, Selumetinib, a MEK inhibitor, is the only currently FDA-approved pharmacotherapy for symptomatic and inoperable PNs in pediatric NF1 patients. The discovery of alternative and additional treatments has been hampered by the rarity of the disease, which makes prioritizing drugs to be tested in future clinical trials immensely important. Here, we propose a gene regulatory network-based integrated analysis to mine high-throughput cell line-based drug data combined with transcriptomes from resected human PN tumors. Conserved network modules were characterized and served as drug fingerprints reflecting the biological connections among drug effects and the inherent properties of PN cell lines and tissue. Drug candidates were ranked, and the therapeutic potential of drug combinations was evaluated via computational predication. Auspicious therapeutic agents and drug combinations were proposed for further investigation in preclinical and clinical trials.

## 1. Introduction

Neurofibromatosis Type 1 (NF1) is a hereditary tumor predisposition syndrome with an incidence of 1 in every 2500 newborns. Approximately half of NF1 patients will develop a benign plexiform neurofibroma (PN), and 15% of PNs transform into a malignant peripheral nerve sheath tumor (MPNST) [1,2]. Because NF1 is a lifelong genetic syndrome, this condition uniquely requires treatments that confer a minimal side effect profile in order to permit long-term or recurrent drug administration [3]. The loss of NF1 potentiates RAS/MEK signaling, which is the hallmark molecular derangement in benign tumors arising from the Schwann cell lineage [4]. Despite the apparent simplicity of this pathoetiology, identifying effective pharmacotherapy has vexed the medical community. Indeed, Selumetinib is the sole FDA-approved drug to treat PNs, and it was not approved until 2019, and only for pediatric patients [5]. New drug targets and drug combinations are urgently needed to treat PNs and potentially inhibit or delay malignant progression in both children and adults. Drug development for rare conditions such as PNs is, however, limited by poor patient accrual, given low numbers of symptomatic tumors as well as geographic dispersion. Computer algorithms that can boost the predictive power of preclinical models are desperately needed to better utilize patient resources and time to develop novel treatments for PNs.

Recent scientific and technological advancements have exponentially expanded the capacity of preclinical drug testing to identify plausible treatments for human diseases; however, these studies rarely translate into novel medications. The challenge of high-throughput preclinical studies to accurately mimic effective human treatments is likely related to the inability of in vitro conditions to approximate the physiologic and molecular milieu of the in vivo tumor microenvironment. Recently, a PN cell line-based drug screening was developed by Ferrer et al., which utilizes several immortalized cell lines tested with the MIPE 4.0 small chemical library [6]. While providing a much-needed platform for drug development, the inherent heterogeneity between different cell lines and the use of non-physiologic conditions in assays can introduce significant variability in the apparent cytotoxic effects of candidate drugs, which undermines downstream experimental verification [7]. This may explain the observation that while multiple drugs inhibit the hyperactive RAS/MEK signaling of NF1 tumor cells in cell culture, most tyrosine kinase inhibitors fail in vivo and in clinical trials [8,9]. So far, Selumetinib remains the most effective and best-tolerated MEK inhibitor in PN patients.

Transcriptome-based gene network analysis is a commonly employed tool for understanding essential molecular signaling within model systems [10,11]. Gene network-based methodology can efficiently identify conserved signaling interactions by identifying co-regulated genes across different model organisms and reveal the correlation between gene networks and biological traits such as disease subtypes and patient survival. It has been employed in the study of glioblastoma [12], non-small-cell lung cancer [13], and osteosarcoma [14]. We aim to use this methodology to reduce background noise in the data and identify salient drug responses across different PN cell lines, and to highlight key molecular pathways that are conserved between cell lines and primary tumors that can be targeted to maximize the success rate of potential drug candidates. Identifying consensus gene networks through this methodology is a valuable tool to better predict efficacious treatments and thus legitimize subsequent validation studies in animals and humans. 

Drug combination therapy serves as a fundamental strategy for cancer treatment in order to improve tumoricidal efficacy while reducing toxicities associated with high-dose drug monotherapy [15,16,17]. Pathway-based vertical drug combinations that target one major signaling cascade at upstream and downstream effectors or horizontal combinations that target parallel signaling cascades simultaneously have been adapted to optimize drug treatment strategies [18,19]. Additionally, combining drugs that are already FDA-approved, or that are actively being tested in clinical trials for other tumor types, may provide therapeutic shortcuts for drug development of rare tumors such as PNs [16]. Recent computational advancements have enabled more eloquent drug combination predictions using experimentally measured high-throughput in vitro monotherapy response data [20,21,22,23]. With a well-planned analytic approach, these methodologies can significantly expand treatment options for rare diseases and prioritize promising combinations for experimental testing. Therefore, we propose a systematic analysis method to mine data from PN drug screening and PN transcriptomes in order to characterize the drug response fingerprint, rank drug candidates according to conserved gene regulation network modules across human PNs and multiple cell lines, and reveal promising drug combinations guided by the molecular drug fingerprints for the treatment of PNs in NF1 patients.

## 2. Materials and Methods

### 2.1. Data and Code Availability

All analyses were performed under the R programming environment. Key packages used include synapser [24], tidyverse [25], dplyr [26], WGCNA [27], ggplot2 [28], enrichplot [29], and ConsensusClusterPlus [30]. The experimental data were provided by the NFhackathon 2020 competition, which were downloaded from https://www.synapse.org (syn4940963 and syn22336443, accessed on 1 October 2020). The code of the NFhackathon project can be found at https://git.io/Jk2iR (accessed on 25 April 2022). 

### 2.2. Drug Screening, PN Cell Lines, and Primary PN Tissue Gene Expression Data

The transcriptome data of PN cell lines were downloaded through NTAP PN Cell Culture Data Portal via syn22351884 [31] and NFhackathon 2020 via syn22336443 (https://www.synapse.org, accessed on 1 October 2020). The drug screening data were contributed by Ferrer et al. using the Mechanism Interrogation PlatE (MIPE 4.0) library [6]. This library comprises 1912 small molecules with different categories. Most of the drugs in this library have known targets, and research has already demonstrated effective tumoricidal activity for specific cancer types. We queried drug information from the ChEMBL database, a manually curated database of bioactive molecules maintained by the European Bioinformatics Institute [32]. The statical term pCHEMBL is unique to this database and is designed to roughly compare efficacy across different molecules by standardizing the half-maximal response concentration, potency, and/or affinity on a negative logarithmic scale. We filtered our drug data using a cutoff of pChEMBL > 6. This cutoff empirically increases the reliability of the curated annotation. The general drug description of the MIPE 4.0 library is downloaded through syn17091507. In total, seven PN-derived cell lines were used, including control cells and multiple tumor lines, and of these, five cell lines had available transcriptome data, which were downloaded via syn22351884, including ipNF05.5 (mixed clone), ipNF05.5 (single clone), ipNF95.11bC, ipNF95.6, and ipnNF95.11c [6,33].

The human primary PN gene expression profiles used in this study were described in Jessan et al. [34]. Transcriptome data of thirteen human primary PN samples were characterized in their study, and the normalized data were downloaded through syn6130081. 

### 2.3. Construction of Conserved Gene Regulation Networks

The gene regulation networks were constructed using the WGCNA package. The standardized gene expression data of five PN cell lines were first filtered by the Mean Absolute Deviation (MAD > 1000) to enrich highly expressed and variable genes and were then merged with the primary tumor transcriptomes to build a scale-free network. A soft-thresholding power of 8 was chosen based on the criterion of approximate scale-free topology [35]. The consensus gene regulation networks between PN cell lines and PN tumor tissues were established as 26 network modules and were labeled randomly using different color names. Nine modules were determined to be conserved, as defined by the Z-summary score >5 according to the package manual. 

### 2.4. Construction of Consensus Drug Clustering

To impartially identify drug clusters with consistently similar transcriptional patterns, we adapted the ConsensusClusterPlus package [30]. Drug clusters were determined according to the correlation coefficients between drug responses and the eigengene of conserved gene regulatory network modules. Parameters were tested according to the package manual.

### 2.5. Drug Combination Prediction

The single drug dose–response data were downloaded from https://www.synapse.org with syn5522627 (accessed on 1 June 2021). For each drug in the screening, drug responses were recorded as the cell viability at eleven different concentrations (µM), which were 0.000780415, 0.007023779, 0.002341244, 0.02107129, 0.063213917, 0.189641751, 0.5689253, 1.706775899, 5.120327696, 15.36098309, and 46.08294931. The two extreme concentrations in the screening, 0.000780415 and 46.08294931, were removed from the predictions. IDAPredict.2drug function package was used to predict the drug combination between DrugCluster pairs [23]. Combinations were ranked according to IDAcombo scores, with predicted drug concentrations. 

## 3. Results

### 3.1. Design Principle and Workflow for Prioritized Drug Candidate Lists

Tumor heterogeneity is one significant challenge when interpreting screening-based drug discovery. The data adapted in this analysis used human immortalized cell lines generated from primary PNs [33]. In addition to inter-individual germline genetic diversity contributing to physiologic differences across the cell lines, these cell lines were genetically modified to ensure that they can be cultivated indefinitely for the purpose of longitudinal drug studies. Immortality was achieved through genetic overexpression of mouse CDK4 and human TERT genes. While it is a common scientific practice to introduce such genetic manipulations in order to immortalize cell cultures, doing so alters the native biologic state and confounds the interpretation of drug responses across different cell lines. Thus, whereas the technique is necessary, it also presents additional challenges for interpreting data and verifying pertinent drug candidates. To tackle this, we identified consensus gene network modules between PN cell lines and primary PN tumors using the WGCNA package in the R programming environment [27,36]. Furthermore, we hypothesized that correlation between drug responses in cell culture and gene expression in preserved gene network modules can elucidate which drugs are more likely to recapitulate tumoricidal activity in follow-up in vivo experiments. In vitro drug cytotoxicity data were then correlated with the gene expression eigengenes of each preserved module. The resulting drugs were then submitted to unsupervised clustering to determine drug clusters that impact similar gene network modules. Drug fingerprints were defined according to the pattern of correlation coefficiency. Candidate List-1 summarizes the top-ranked candidates from each drug cluster.

**Figure 1:**
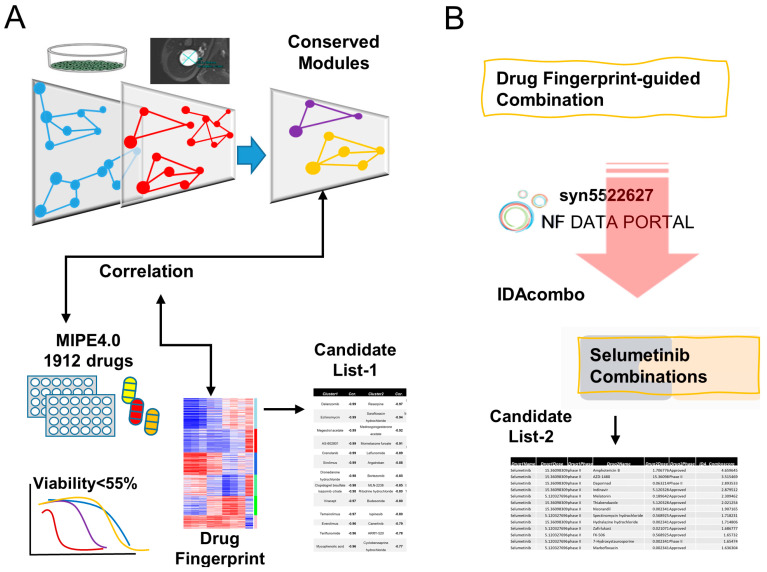
Analysis pipeline to prioritize candidates and drug combinations. (**A**) Conserved gene expression network modules were constructed by cross-referencing the transcriptomes of immortalized human PN cells against resected PN tumors. Drugs that reduced the median viability of cell lines by more than 55% from the MIPE 4.0 library were selected for further analysis. Correlations were then determined between the conserved gene modules and drug responses. Drugs were clustered according to these correlations to generate a drug response fingerprint. The drugs in each cluster were then limited to those with FDA approval or being actively studied in clinical trials (Candidate List-1) (**B**). Both drug fingerprint and the IDAcombo algorithm were used to predict drugs that are likely to be complementary to Selumetinib in Candidate List-2.

Drug combinations were evaluated by the IDAcombo package [23]. This algorithm relies on the principle of independent drug activation (IDA), which assumes that the expected effect of a combination of non-interacting drugs is best defined by the more efficacious drug in the combination. Whereas individual cell lines exhibit different sensitivities to the same drug combination, the overall average response across multiple cell lines is almost always greater than that of monotherapy because each cell line is proffered two or more drugs to which it may respond [37]. We predicted combinations with the guidance of drug fingerprints, and the promising drug combinations with Selumetinib were further prioritized as the Candidate List-2. The workflow utilized is demonstrated in Figure 1.

### 3.2. Preserved Network Modules Reveal Biological Consistencies across PN Models

**Figure 2:**
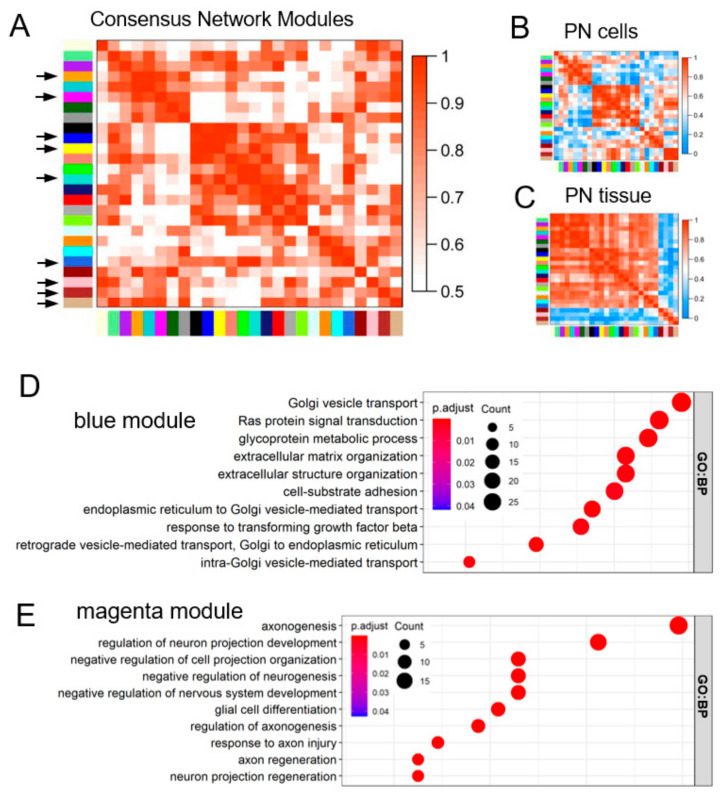
Preserved network modules in PN cells and primary tumors. (**A**–**C**) Eigengene adjacency demonstrated the preserved gene network modules (indicated by black arrows in (**A**)) from PN cells (**B**) and tumor tissue (**C**). (**D**,**E**) Gene ontology enrichment analysis of preserved blue module (**D**) and magenta module (**E**) under the biological process category (GO:BP).

The transcriptomes of cell lines used in the drug screening and the untreated surgically-resected primary PN from Jessen et al. were downloaded through the NF Data Portal [34]. To identify the consensus gene regulatory network modules among PN cells and primary tumor tissue, we constructed a block-wise network using the WGCNA package (see Methods, Supplementary Figure S1). Twenty-six network modules were defined with various module sizes and preservation as Z-summary scores (Supplementary Table S1). Modules were labeled with different color names, and the module eigengene was defined as the first principal component of each module. The eigengene adjacency value codified the consensus preserved gene network modules from both PN cells and tumor tissue (Figure 2A–C). The modules with Z-summary scores of more than 5 were selected as preserved modules among the different PN models, which included brown, blue, magenta, tan, pink, turquoise, salmon, yellow, and midnight-blue modules (hereafter referred to as “preserved modules”, indicated with arrows in Figure 2A). 

To interpret the biological features in preserved modules, we performed gene ontology (GO) analysis. The top enriched GO terms from the biological process category (GO:BP) in each conserved module are listed in Supplementary Table S2. The highly enriched GO:BP terms of blue and magenta modules were shown in Figure 2D,E. The “Ras protein signal transduction” term is highly associated with the blue module, reflecting the cardinal feature of NF1-associated PNs. Interestingly, the magenta module is strongly enriched in a series of terms describing development regulation of the peripheral nervous system and interactions between neurons and Schwann cells. Specifically, in magenta, a subset of neural crest progenitor markers, such as NES, SOX10, ERBB3, and NGFR, were reported as highly expressed markers of Schwann cell progenitors and the signatures of stem-like tumor cells in NF1 tumors [4,38]. Genes in different preserved modules are summarized in Supplementary Table S3. 

These results are consistent with the known pathoetiology of PNs that (1) loss of function of the NF1 gene potentiates the Ras pathway signaling [39] and (2) Schwann cell progenitors can serve as the cell of origin of PN and subsequently recruit NF1-heterozygous non-neoplastic cells into the tumor microenvironment [38]. The preserved modules from unsupervised analysis independently recapitulate PN tumor biology, providing validation for using this method to direct drug selection for further study. 

### 3.3. Preserved Module-Based Drug Fingerprints and Candidate List-1 

**Figure 3:**
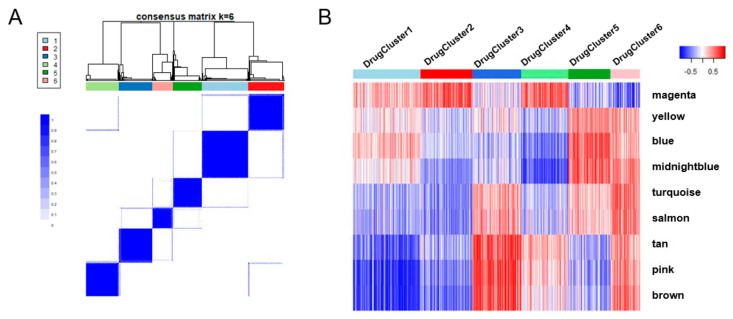
Consensus clustering analysis using the correlations between drug responses and eigengenes of preserved modules. (**A**) The consensus clustering plot revealed the 6 clusters. (**B**) The heatmap demonstrated unique patterns as drug fingerprints using correlations between the drug responses and preserved modules.

Drug responses in the screening were downloaded from the NF Data Portal. We preselected drugs by setting a cutoff of cell viability <55% for the inhibitory median response across PN cell lines. The inhibitory median response is defined by the area under the curve, reflecting the viability of the cells in the screening. This filter yielded 1831 drug candidates from 1912 oncology-focused compounds within the MIPE 4.0 library. The Pearson correlations between the eigengene of each preserved module and the response to each preselected drug were calculated, as demonstrated in a heatmap in Supplementary Figure S2. We then performed consensus clustering analysis on those drugs according to the correlation coefficiency (see Methods) and identified six drug clusters (Figure 3A). For each drug, the correlation coefficients related to each PN preserved network module serve as a unique drug fingerprint relevant to the biology of PNs (Figure 3B).

In the MIPE4.0 library, 20% of drugs are currently FDA-approved, and 40% are undergoing active testing in clinical trials (2019 annotations). Limiting drug candidates to these two categories will allow for more immediate translation of our findings to clinical trials as the human safety and tolerability data are already defined. We filtered drug candidates by annotated statuses of FDA-approved, Phase II, and Phase III (advanced status hereafter) in each cluster and ranked these drugs according to the negative correlation between a given drug and any conserved module. Negative correlations imply that the genes in each module are downregulated with the increased drug concentration and can thus serve as potential treatment targets [40]. The top 15 drugs in each cluster that satisfied these criteria are listed in Table 1, and the completed Candidate List-1 can be found in Supplementary Table S4. Selumetinib was identified in Candidate List-1 DrugCluster 4, supporting the validity of our analysis strategy.

### 3.4. Drug-Fingerprint-Guided Combination Strategies

**Figure 4:**
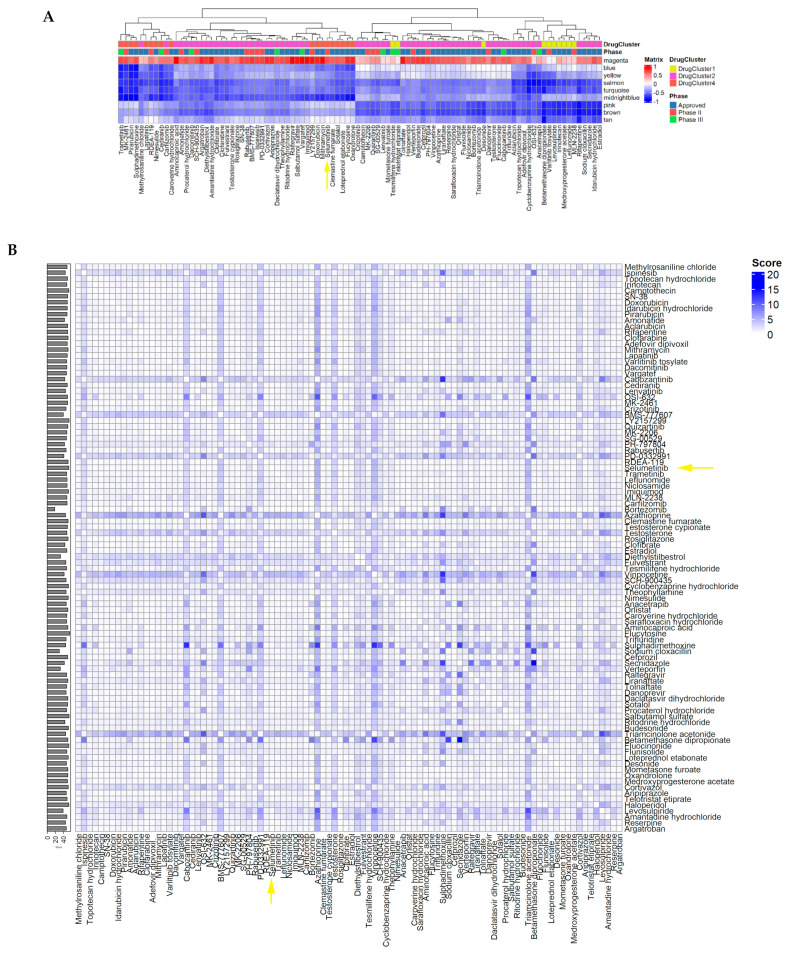

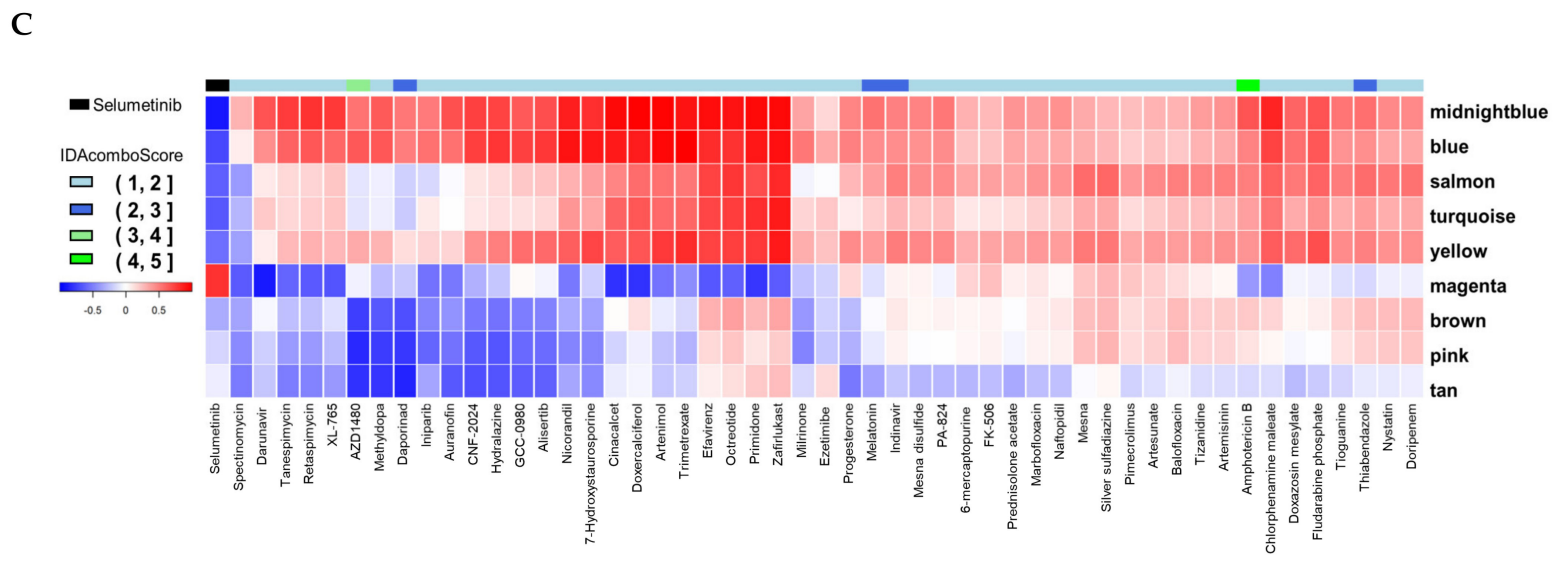
Fingerprint-guided drug selection and combination strategies. (**A**) Selumetinib-like drug candidates with the similar fingerprint pattern. The color bar at the top of the figure indicates the corresponding drug clusters and clinical status of each drug. Selumetinib was indicated by the yellow arrow. (**B**) The heatmap of IDAcomboscore of any two-drug combination among the Selumetinib-like drug candidates. (**C**) Complementary pattern between Selumetinib and selected drugs from DrugCluster5. Each column is comprised of a single drug from DrugCluster 5. The color bar at the top of the figure indicates the IDAcombo score. Each cell is colored according to the Pearson correlation between the drug responses and the eigengene of each preserved network module. The best predicted drug combinations with Selumetinib include JAK1/2 inhibitor AZD-1480, antibiotic Amphotericin B, nicotinamide salvage pathway inhibitor Daporinad, circadian hormone melatonin, antiviral indinavir, and anti-parasitic thiabendazole.

The traditional evaluation of drug combinations is labor-intensive, time-consuming, and expensive, considering the numerous possibilities [41]. With the accessibility of drug screening data, applying computational prediction models to identify drug combinations offers an attractive alternative. Multiple methods have been utilized in predication, such as machine learning, drug similarity, and/or drug interactome [42]. With the drug fingerprints developed in this study, we were able to cluster the drugs, reflecting the corresponding biological relevance. A similar fingerprint pattern may suggest the common signaling cascade on the molecular level. Identification of Selumetinib-like drugs or combining Selumetinib with other candidates may provide more options to increase treatment efficacy, minimize toxicity and prevent drug resistance. The vertical combination strategy combines drugs targeting the same biological process or pathway, which has been adapted to minimize drug resistance mechanisms [43]. With biological relevance indicated by drug fingerprints, we propose to seek the drug candidates in the screening with a Selumetinib-like fingerprint and evaluate the vertical combination efficacy among the candidates. Selumetinib was localized in DrugCluster 4 and demonstrated negative correlations to all conserved network modules except for magenta (Figure 4A). Drug candidates that have advanced clinical status and demonstrate similar fingerprints as Selumetinib are enriched in DrugCluster1, DrugCluster2, and DrugCluster4 (Figure 4A). 

To survey the combination potentials among the Selumetinib-like candidates, we adapted the two-drug combination algorithm from IDAcombo package, which predict the efficacy based on the single drug dosage–response data. The important features of this methodology are that (1) the predictions are concentration-dependent and represent an average response across populations of cell lines, which mimic the efficacy evaluation in clinics and serve as guidance for experimental verification; (2) it scores the combinations to prioritize the predicted combinations [23]. The predicted IDAcomboscores are shown in the heatmap (Figure 4B). The detailed drug responses among the PN cell lines, known mechanisms, and targeted genes are summarized in Supplementary Table S5. 

Analogous to the horizontal combination strategy, combining drugs with complementary fingerprint patterns may provide a therapeutic advantage by targeting different biological processes, such as DrugCluster 1 and 3, DrugCluster 2 and 6, and DrugCluster 4 and 5, demonstrated in the drug cluster fingerprints (Figure 3B). Selumetinib was located in DrugCluster 4, which has a drug fingerprint pattern that is complementary to DrugCluster 5. Therefore, we predicted the combination efficacy of Selumetinib and partner drugs from DrugCluster 5, which may direct salient treatment combinations for subsequent clinical trials. For those combinations with an IDAcombo score >1, their drug fingerprints and combination scores were demonstrated as color bar in the drug fingerprint heatmap (Figure 4C). Combinations between Selumetinib and DrugCluster 5 and all the predicted combinations from the above complementary drug clusters were summarized in Supplementary Table S5.

## 4. Discussion

The current study employs a systems-down approach to drugs and drug combinations to treat NF1-associated PNs. Starting with the transcriptomic results and a high-throughput PN cell culture drug assay, we detected co-regulated network modules and identified drug clusters with similar fingerprints according to the conserved network modules. There are several advantages to employing this computational methodology to select drugs and drug combinations for further preclinical and clinical testing. The first is that a rational and quantifiable line is drawn between the preclinical model and the human tumor. By their nature, cell culture methodologies isolate cancer cells from wildtype tumor support tissues, train them to grow upon a non-biologic 2-dimensional matrix, and submit them to frequent traumatic dissociation and centrifugation, which can favor genetic drift and lead to de novo physiologies and behaviors that distinguish them physiologically from the original tumor. This can explain why so many promising cell-culture assay results are not borne out in clinical trials. The methodology highlighted in this study uniquely focuses only on transcriptionally conserved gene networks across both cultured cells and primary PNs, thereby filtering out drugs with effects that are less likely to be replicated in vivo.

The current study investigates an integrated computational method to vet salient drugs that merit evaluation in clinical trials based upon the transcriptional effects and cytotoxic profile of a high-throughput cell culture assay. Safety and toxicity are of paramount importance in this patient population that has the propensity for requiring lifelong or recurrent treatment. Understandably, clinical judgment must also be employed in determining which drugs and drug combinations would be worthwhile to investigate further in vivo and in human trials. For example, while Melatonin together with Selumetinib had a high IDAcombo score, Melatonin together with chemotherapy has been tested in lung, breast, and ovarian cancers with minimal added benefit [44]. Drugs such as Thiabendazole and Amphotericin B, which are indicated for parasitic and bacterial infections, respectively, likely have too high a side effect profile for chronic or recurrent use in NF1. Daporinad has a very interesting mechanism of action (inhibition of nicotinamide salvage pathway); however, its intravenous route of administration and prolonged infusion time may prove an impediment to patient care. The oral small molecule ATP-competitive JAK2/3 inhibitor AZD-1480 would be a logical drug to investigate further with Selumetinib due to the parallel and synonymous activity of JAK/STAT and the RAS/MEK signaling in promoting cell mitosis [45,46]. 

JAK activates STAT signaling, which transcriptionally represses the epigenetic regulator ARID1B. In Schwann cell progenitors, the JAK/STAT pathway is important in autoimmune, and β-catenin regulates the maintenance of stem-like pluripotency, whereas the concurrent loss of NF1 in genetically engineered mouse models leads ultimately to neurofibroma generation and maintenance myeloproliferative disorders, and drugs targeting this pathway are currently used to treat rheumatoid arthritis, ulcerative colitis, and myelodysplastic syndrome.

JAK2 signaling is implicated in neurofibromagenesis [47]. JAK/STAT signaling within myelocytes present in the tumor microenvironment may also contribute to tumor formation by activating pro-mitotic paracrine signaling [48]. Molecularly, JAK activates STAT signaling, which transcriptionally represses the epigenetic regulator ARID1B, leading ultimately to neurofibroma initiation and maintenance. Of note, neither of the JAK/STAT pathway inhibitors employed in the above studies were included in the MIPE 4.0 drug list, including inhibitor XAV-939, a stabilizer of the beta-catenin decretory molecule, and pan-JAK/Tyk2 inhibitor FLLL32.

Clinically, AZD-1480 failed to achieve safety endpoints or clinical benefit in advanced solid tumors [49]. While AZD-1480 alone is unlikely to provide a clinical benefit in humans due to adverse side effects, a focused in vitro and in vivo assessment of AZD-1480 together with Selumetinib would be valuable to determine whether dual targeting of these two pathways could be achieved with lower drug doses and could provide proof of principle for the methodology proposed in this study. Also included in the MIPE 4.0 library are JAK/STAT inhibitors Cucurbitacin I (not FDA approved), TG-46 (not FDA approved), Degrasyn (currently being tested in a Phase III clinical trial), and Tofacitinib (FDA approved), each of which achieved an IDACombo score of >1 alongside Selumetinib (Supplementary Table S5). Other drugs targeting the Jak/Stat pathway that have been developed more recently than the MIPE 4.0 drug library could also be investigated, such as Ruxolitenib—a JAK1/2 inhibitor that is FDA approved for the indication of myelofibrosis and topically for atopic dermatitis. Additionally, oral investigational agent Pelabresib (CPI-0610) is a bromodomain and extra-terminal domain (BET) protein inhibitor undergoing concurrent testing with Ruxolitenib in phase 3 clinical trial for myelofibrosis therapy. Other FDA-approved JAK inhibitors for rheumatoid arthritis include Baricitinib and Fedratinib. Testing these drugs alone and in combination with Selumetinib would provide a proof of principle justification for utilizing this high throughput assay and analysis to identify clinically salient conjugate drug combinations to treat plexiform neurofibromas. A focused preclinical analysis of co-administration of these JAK/STAT targeted agents with Selumetinib +/− other MEK inhibitors is warranted. Future investigations can include preselecting gene modules of interest and investigating drug combinations with recommended dosing regimens using the IDAcombo score. Among the conserved network modules in this study, the blue module recapitulates abnormal RAS signaling among the PN cells and primary tumors, demonstrating the robustness of our methodology. Our recent publication on NF1 tumor cell-of-origin revealed a stem-like tumor cell population in both mouse models and the primary human atypical neurofibromas [38]. Interestingly, the magenta module highly enriched the signature genes for stem-like tumor cells, which is consistent with cancer stem cell theory that argues stem-like tumor cells originate from neural crest stem cells and serve as a constitutive force to drive NF1 PN tumorigenesis [4,38,50]. Further analysis of this module may help to identify PN cancer stem cell-specific drug targets for treatment.

Phenotype-based drug screening significantly extends the drug candidate pool, especially for diseases with limited treatment options. Computational models have previously been developed to identify promising drug candidates and combinations in the drug screening for anti-tumoral treatment [40,51,52,53]. However, the limitations of cell culture models and predictive drug selection algorithms remain an impediment to the generation of biologically valid predictive results. To adjust for these limitations, our approach of integrating network-based biologic models and combining heterogeneous data sources could represent a breakthrough in identifying promising drug candidates or combinations [54]. In the current study, post hoc supervised data trimming was employed to select Phase II or Phase III clinical trials or FDA-approved medications. While the current study was intentionally limited to clinically vetted compounds to accelerate bench-to-bedside translation to an underserved disease population, this method could alternately be used in the future to test experimental drugs and drug combinations to explore novel treatment avenues in preclinical and Phase I trials for NF1 patients with PNs.

## 5. Conclusions

Our study discovered the gene regulation network modules conserved in PN tumor cell lines and primary PNs, and the stem cell-like gene network module was identified, which is consistent with the mouse model study. The conserved network modules prioritize drug candidates in the PN cell line-based drug screening. Drug combinations were proposed according to the computational analysis for further preclinical and clinical testing in NF1-associated PNs. 

## Figures and Tables

**Table 1:** The top 15 candidates in each drug cluster.

Cluster1	Cor.	Cluster2	Cor.	Cluster3	Cor.	Cluster4	Cor.	Cluster5	Cor.	Cluster6	Cor.
Delanzomib	−0.99	Reserpine	−0.97	Prostaglandin E2	−0.87	Ramipril	−0.98	Darunavir	−0.89	Diphenhydramine hydrochloride	−0.97
Echinomycin	−0.99	Sarafloxacin hydrochloride	−0.94	Methylprednisolone	−0.77	Cortivazol	−0.98	Daporinad	−0.85	Carisoprodol	−0.95
Megestrol acetate	−0.99	Medroxyprogesterone acetate	−0.92	Indapamide	−0.75	Nitisinone	−0.95	BMS−707035	−0.84	Naratriptan hydrochloride	−0.95
AS−602801	−0.99	Mometasone furoate	−0.91	Eflornithine Hydrochloride	−0.74	Nimesulide	−0.95	AZD−1480	−0.83	Trazodone hydrochloride	−0.94
Crenolanib	−0.99	Leflunomide	−0.89	Moroxydine	−0.71	Enoxolone	−0.95	Zidovudine	−0.81	Sulindac	−0.94
Sirolimus	−0.99	Argatroban	−0.88	Linezolid	−0.70	Aniracetam	−0.94	Cinacalcet hydrochloride	−0.80	Rilmenidine	−0.94
Dronedarone hydrochloride	−0.98	Bortezomib	−0.85	Crotamiton	−0.70	Fluocinonide	−0.93	Doxercalciferol	−0.79	Torasemide	−0.90
Clopidogrel bisulfate	−0.98	MLN−2238	−0.85	Diclofenamide	−0.70	Ethosuximide	−0.92	Methyldopa	−0.78	Calcitriol	−0.90
Ixazomib citrate	−0.98	Ritodrine hydrochloride	−0.80	Thiamphenicol	−0.69	Betamethasone	−0.92	Primidone	−0.78	Tolvaptan	−0.88
Viracept	−0.97	Budesonide	−0.80	Varespladib	−0.69	Diindolylmethane	−0.91	Atazanavir sulfate	−0.78	Fluvoxamine maleate	−0.87
Temsirolimus	−0.97	Ispinesib	−0.80	Tariquidar	−0.69	Clobetasol propionate	−0.91	Tipifarnib	−0.77	Veliparib	−0.86
Everolimus	−0.96	Canertinib	−0.79	Monatepil	−0.67	Telotristat etiprate	−0.91	Ethisterone	−0.77	Indomethacin	−0.83
Teriflunomide	−0.96	ARRY−520	−0.78	Indiplon	−0.67	Aliskiren hemifumarate	−0.90	Formestane	−0.74	CAL−101	−0.83
Mycophenolic acid	−0.96	Cyclobenzaprine hydrochloride	−0.77	Nepicastat hydrochloride	−0.66	Betamethasone valerate	−0.90	Ritonavir	−0.73	Sinomenine	−0.83
Daunorubicin	−0.95	Mithramycin	−0.76	Lubiprostone	−0.65	Selumetinib	−0.90	AT−13387AU	−0.72	Flurbiprofen	−0.82

## Data Availability

All data were downloaded from the public online databases with administrative approval accordingly. The analysis codes are available through synapse.org via Synapse ID: syn24317288 and syn26004582.

## Supplementary Materials

The following supporting information can be downloaded at: https://www.mdpi.com/article/10.3390/brainsci12060720/s1, Figure S1: Scale-free network building to identify the conserved gene regulation network modules; Figure S2: The heatmap shows the correlation coefficient between the conserved gene network modules (row) and drug responses (column). Supplementary Table S1: Z-summary scores for gene network modules. Supplementary Table S2: GO analysis of genes in conserved network modules. Supplementary Table S3: Gene symbols in each conserved network modules. Supplementary Table S4: Candidate List-1. Supplementary Table S5: Candidate List-2.
