# Supplementary figures and images for: Integrated Drug Mining Reveals Actionable Strategies Inhibiting Plexiform Neurofibromas
Source: Brain Sci. 2022 May 31;12(6):720. doi: 10.3390/brainsci12060720 (PMC9221468; doi:10.3390/brainsci12060720)

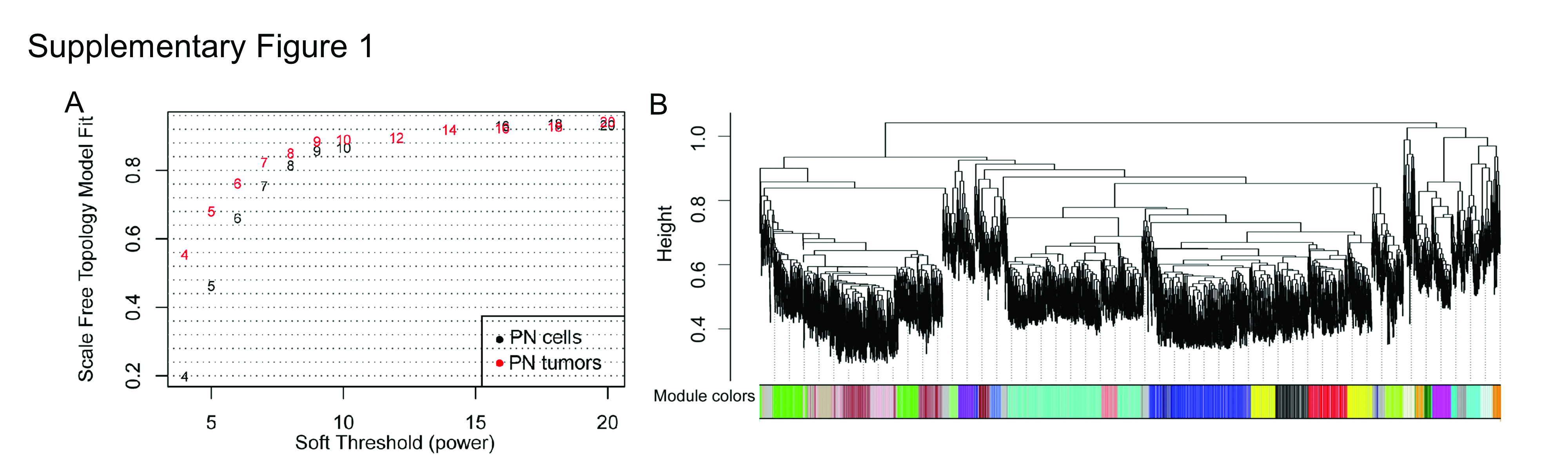

Supplement: Supplementary file 1 [file brainsci-12-00720-s001.zip › Supplementary Figure S1.tif]
